# Supplementary material for: Prevalence and associated factors of occupational injuries in an industrial city in Ghana
Source: PLoS One. 2024 Mar 28;19(3):e0301339. doi: 10.1371/journal.pone.0301339 (PMC10977681; doi:10.1371/journal.pone.0301339)
Supplement: S1 Appendix — (DOCX) [file pone.0301339.s001.docx]

Tema municipality

(25 communities)

Eight communities

(Communities 1, 2, 3, 4, 7, 8, 9, and 10)

Households (proportional allocation)

Study participants (everybody who meets the inclusion criteria)

Random sampling of communities

Random sampling of communities

Sampling of study participant

Appendix 1: Sampling approach of study participants
