# Supplementary material for: Prevalence and associated factors of occupational injuries in an industrial city in Ghana
Source: PLoS One. 2024 Mar 28;19(3):e0301339. doi: 10.1371/journal.pone.0301339 (PMC10977681; doi:10.1371/journal.pone.0301339)
Supplement: S2 Appendix — (DOCX) [file pone.0301339.s002.docx]

**Appendix 2: Multi-collinearity test results**

| **Variables** | **VIF** | **Tolerance** | **R – Square** |
| --- | --- | --- | --- |
| Age group | 2.11 | 0.4734 | 0.5266 |
| Sex | 2.05 | 0.4875 | 0.5125 |
| Gender | 1.32 | 0.4734 | 0.5266 |
| Educational level | 2.13 | 0.4689 | 0.5311 |
| Type of engagement | 1.12 | 0.8943 | 0.1057 |
| Monthly income | 2.06 | 0.4864 | 0.5136 |
| Industry type | 1.24 | 0.8044 | 0.1956 |
| Health and safety training | 1.64 | 0.6095 | 0.3905 |
| Satisfaction with health and safety measures at the workplace | 1.24 | 0.8041 | 0.1959 |
| PPE use | 1.36 | 0.7375 | 0.2625 |
| Mean VIF | 1.63 | | |
