# Supplementary material for: Prevalence and associated factors of occupational injuries in an industrial city in Ghana
Source: PLoS One. 2024 Mar 28;19(3):e0301339. doi: 10.1371/journal.pone.0301339 (PMC10977681; doi:10.1371/journal.pone.0301339)
Supplement: Attachment — Submitted filename: RESPONSE TO REVIEWERS.pdf [file pone.0301339.s005.pdf]

ALLEN CLINIC
FAMILY HEALTHCARE SERVICES
P. O. Box X543 FNT, Kumasi, Ghana
Tel: 0322493429 | 0507171650
Loc: Ahensan Estates near KNUST Gate, Kumasi
Email: allenfhs@gmail.com

RESPONSES TO REVIEWERS' COMMENTS – EPIDEMIOLOGY OF OCCUPATIONAL INJURIES IN AN INDUSTRIAL CITY IN GHANA: A CROSS-SECTIONAL SURVEY *(MANUSCRIPT ID.* PONE-D-23-10198).

17th June 2023

The Editor-In-Chief
PLOS One

Section of Paper	Reviewers' Comments	Response from Authors
**Academic Editor**		
**Methodology and Study Design**	Provide a more detailed description of the study design, including the sampling method employed and the rationale behind it. This will enhance the transparency and reproducibility of your study.	This has been done as suggested.<br><br>This was a community-based cross-sectional survey among workers in the Tema Metropolis, an industrial setting in Ghana from 22nd December 2020 to 27th February 2021. The adoption of this study design is justified by its ability to estimate both prevalence and inferential statistics like odds ratio which shows the strength of association between dependent (occupational injuries) and independent (age, sex, type of engagement etc.) variables (28). [Page 4, Lines 110-113]
	Clearly define the inclusion and exclusion criteria for selecting participants. This will help readers understand the population under investigation and improve the study's generalizability	This was present in the initial submission but has been revised to improve clarity. The revised section now reads;<br><br>The selection of participants into the study was strictly based on satisfying the inclusion criteria which was being between 20 to 60 years old and having been working in Tema for at least twelve months. All workers in administrative positions (such as receptionists, accounting staff and secretaries) were excluded from the study. [Page 5, Lines 124-127]
	Elaborate on the data collection process, including the tools used, the training provided to data collectors, and the steps taken to ensure data quality and minimize bias.	These were provided initially but have now been made more explicit and clearer.<br><br>Data collection was done using a structured questionnaire running on a smartphone with the aid of Kobo Collect.

		The questionnaire was self-developed entirely new by the investigators through a literature review [10,21,23,33–37]. Data was collected on socio-demographic variables including age, sex, educational level, monthly salary, type of engagement, health and safety practices such as usage of PPE, frequency of PPE use and access to health and safety training and occurrence of occupational injuries with emphasis on the types, numbers and mechanisms of the injuries. The usage of PPE was measured by asking participants whether they wore PPE at the workplace when performing a task with a 'yes' or 'no' response while the frequency of PPE usage was assessed by asking participants how often they wear the PPE when performing a task at the workplace with 'always' or 'sometimes' response. 'Always' means that the worker wears PPE anytime he or she is performing a task while 'sometimes' means that the worker only wears the PPE as and when he or she deemed it fit (i.e. not wearing it all times when performing a task at the workplace. Occupational injury was operationalized as all injuries sustained while performing a task at the workplace as defined by the International Labour Organization [38]. To avoid misclassification of occupational injury, study participants who reported sustaining work-related injuries were asked to describe how and where the injury occurred.<br>The questionnaires were administered in a face-to-face interview in English or the local Twi language by four trained research assistants. The research assistants were all final-year post-graduate Occupational Health and Safety students from the School of Public Health, Kwame Nkrumah University of Science and Technology, Ghana with previous experience in quantitative data collection. They were trained for 3 days by an expert in quantitative data collection and a biostatistician on community entry, consent, the use of Kobo Collect, the selected sampling technique as well as uniform translation of the questions into the local Twi language to ensure consistency in the data collected. The questionnaire was pretested among 50 workers in the North Industrial Area enclave in Accra which is 36 km away from Tema. Based on the feedback from the pre-test, the study tools were modified to suit the objectives of the study before it was used in the main study.<br>[Page 6, Lines 147-173]

**Statistical Analysis**	Describe the statistical methods employed in analyzing the data more comprehensively. Specify the statistical tests used, including appropriate adjustments for confounding variables and potential sources of bias. Include details on the software used for statistical analysis, along with the version number, to facilitate reproducibility.	We performed the analysis again using a logistic regression analysis with a backward stepwise approach. This has been provided in the revised manuscript. The specific section now reads as;<br><br>Data was cleaned by checking for wrong entries and exported to Stata version 16.0 (StataCorp, College Station, USA) for analysis. Summary statistics were conducted and presented as means with standard deviations for continuous variables, and frequencies and percentages for categorical variables. Associations between predictor (such as age, sex, health and safety training etc.) and outcome (occupational injury) variables were explored using either Chi-square or Fisher's exact (for proportions with subgroups <5) tests. The primary outcome was a self-reported occupation-related injury in the twelve months prior to the interview. Bivariate and multivariate logistic regression analyses were employed to quantify the strength of association between the occurrence of occupational injuries and their determinants. The effects of other covariates on the outcome were adjusted for in the final model using a backward stepwise approach with a p-value of 0.1. In the multivariate logistic regression model, all predictor variables that had both statistically significant and insignificant association with the outcome variables were taken into account. They were considered important or potentially confounding based on previous studies (20,23,33,34,37,39) in arriving at the final backward stepwise regression model. For the bivariate and the final multivariate models, statistical significance level was kept at a p-value of <0.05 and odds ratios presented at a 95% confidence level. [Page 7, Lines 174-190]
**Results and Discussion**	Present the results in a clear and organized manner, using appropriate tables and figures to enhance the readability and interpretation of the findings.	This has been done as suggested.
	Discuss the limitations of the study, including any potential biases or confounding factors that may have influenced the results. This will provide a more comprehensive understanding of the implications of your findings.	We controlled for confounding variables in the analysis by adjusting for these variables in the logistic regression analysis. This has been acknowledged in the revised manuscript under subheading ''Data management and statistical analysis''. Again, to reduce information bias, participants were asked to describe how and where the injury occurred and this has been acknowledged in the revised manuscript under subheading ''Data collection'' [Page 6]

		Other limitations have been provided under the subheading ‘’strengths and limitations of the study’’ [Page 15, Lines 342-354]
	Compare your results with existing literature on occupational injuries in similar settings to contextualize your findings and highlight the novel contributions of your study.	We compared our findings to a study in Ethiopia which was also conducted among workers in an industrial city in the initial manuscript. In the revised manuscript, we have also compared the findings in the present study to similar studies conducted among workers in industrial city in India. It reads;<br><br>However, in an urban city in India, Sashidharan et al (2017) in a study among industrial workers reported an injury prevalence of 44.3% (33) which is lower than the prevalence rate (63.4%) observed in our study. In the present study, we estimated injury prevalence among nine different occupational groups while the study in India (33) estimated injury prevalence among two different occupational groups which could affect the injury rates in the two studies. Despite that, all these studies show a high prevalence of occupational injuries among workers. [Page 13, Lines 271-277]
**Conclusion**	Revise and strengthen the concluding statements to accurately reflect the key findings of your study.	This has been done as suggested. The revised section now reads;<br><br>There is a high prevalence (64.7%) of occupational injuries among the study participants. Factors associated with this are employees’ level of education, type of engagement, type of industrial work, health and safety training and their level of satisfaction with health and safety measures at the workplace. It is imperative for effective mitigation measures to be instituted for this high-risk population. These include training and retraining in health and safety, education and awareness campaigns, as well as policy enforcement measures among workers, especially, those with a low level of education and temporary staff. [Page 13, lines 356-366]
	Clearly state the implications of your research and highlight potential avenues for future research and intervention strategies	This has been done as suggested. It reads;<br><br>The present study provides useful information that can guide efforts in the promotion of health and safety among workers at the workplace. Future studies can adopt a qualitative approach to explore the satisfaction of health and safety policies at the workplace. [Page 13, Lines 363-366]

Language and Clarity	Carefully proofread the manuscript to address grammatical errors, awkward phrasing, and typographical mistakes.	Please, this has been done as suggested
	Ensure consistency in terminology and abbreviations throughout the manuscript	Please, this has been done as suggested
**Reviewer 1**		
	I appreciate the invitation to review the manuscript. The manuscript was reviewed and general comments about the subject and content of paper was presented as follow. Based on our review, it seems that the mentioned paper is not suitable for publication in PLOS ONE.<br><br>• The content of paper is not enough for publication in PLOS ONE<br>• It is not observed new finding in this research.	This study contributes to the existing body of knowledge on the epidemiology of occupational injuries in Ghana among workers from different occupational background. Particularly, Ghana is in the process of coming up with a single health and safety policy to promote health and safety at the workplace. This study provides useful information that can inform policy direction.<br><br>Secondly, no study has been conducted in a major industrial city in the West African subregion, particularly Ghana, that estimates injury prevalence and determinants. This is a gap considering that the type of hazards that a worker in a non-industrial setting is exposed to may be different. Hence, it is essential to estimate the burden of injury and their contributing factors.
**Reviewer 2**		
Introduction	Page 3, line 61<br>"The global burden of occupational injuries is estimated to be 1.9 million deaths in 2019" is this data/figure includes occupational diseases OR occupational injuries only?	It includes diseases and this has been acknowledged in the revised manuscript. It now reads:<br><br>The global burden of occupational injuries and diseases is estimated to be 1.9 million deaths in 2019. [Page 3, lines 60-61]
	Pages 4, line 88-92<br>The statement is about the associated factors; as result, it is better to merge with the previous paragraph.	This has been done as suggested. Thank you for your suggestion. [Pages 3,4, Lines 83-87]
	Pages 4, line 93:<br>"Apart from the few studies mentioned above,…" What your study added to these few studies?	This has been provided in the revised manuscript.<br><br>Apart from the few studies mentioned above, little is known about the epidemiology of occupational injuries among workers in a major industrial city with over five hundred companies and has the largest Port in Ghana. These studies only focused on estimating injury prevalence among specific working populations such as general construction workers (24), small-scale gold miners (22), healthcare workers (26), solid waste collectors (25) and Emergency Medical Technicians (27). This study estimated injury prevalence and their

		determinants among workers in different occupational groups (such as Port and Harbour, Manufacturing Companies, Tourism and Hospitality, Pharmaceuticals, among others). Estimating injury rates for the different occupational groups will give us a snapshot of their burden to inform policy direction towards an appropriate preventive strategy. This will help to improve the health and safety of workers in their working environment in Ghana and beyond. Continually measuring injury prevalence and its associated factors among workers in an industrial city will help to strengthen the body of evidence that policy makers, health and safety managers and other key stakeholders need to guide occupational health and safety. [Page 4, Lines 92-105]
**Methods:**	Pages 5, line 123<br>Why you use 1.2 as design effect? Why not 1.5 or 2? Could you support with credible source/evidence	Thank you very much for your careful and meticulous observation on our manuscript. We have accepted your insightful observation on the sample size estimation relating to the design effect; the use of a design effect of 1.2 instead of 1.5. We acknowledge that this was an error and the necessary corrections have been effected in the manuscript. We apologize and will make sure that due diligence will be carried out in our future submissions. [Page 5, Line 131]
	Pages 6, line 150-152 "'Occupational injury' was operationalized as all injuries sustained while performing a task at the workplace that resulted in a loss of at least 12 hours of productive time" How did you confirm the impact of injury (a loss of at least 12 hours of productive time)?	We used the International Labour's definition of occupational injury to classify an injury as an occupational-related. To avoid misclassification of the outcome, participants who reported an occupational injury were asked to describe how the injury occurred. This has been duly recognized in the revised manuscript. This now reads as;<br><br>'Occupational injury' was operationalized as all injuries sustained while performing a task at the workplace as defined by the International Labour Organization (30). The study participants who reported sustaining an occupational injury were asked to describe how the injury occurred to avoid misclassification. [Page 6, Lines 159-162]
	Pages 6, line 160-161 Are you used Chi square or Fisher's exact tests analysis? Better to state what you already used	We used either Chi-square or Fisher's exact (for proportions with subgroupings <5) tests. This has been acknowledged in the revised manuscript<br><br>Associations between predictor (such as age, sex, health and safety training etc.) and outcome (occupational injury) variables were explored using either Chi-square

		or Fisher's exact (for proportions with subgroups <5) tests. [Page 7, Lines 178-180]
**Results**	Pages 7, line 185-186 "The proportion of study participants that experienced an occupational injury in the last 12 months was 64.7%." State the proportion of occupational injuries with 95% CI; the lower and upper confidence intervals	This has been done as suggested. The section in reference now reads as;<br>The proportion of study participants that experienced an occupational injury in the last 12 months was 64.7% (95%CI: 0.60 – 0.69). [Page 9, Lines 217-218]
	Pages 8, line 197-199 "Over 54.9% of them were supplied with PPE and 77.3% of the study participants indicated that they used PPE at the workplace" How the proportion of PPE use is much higher than the proportion of PPE supply (77.5% vs 54.9%)	Once again, this was an error which has been corrected in the revised manuscript. We are very grateful for drawing our attention to it. The revised section now reads;<br><br>Over 22.7% (104) of the workers indicated that they used PPE at the workplace. Out of the 104 workers that indicated they used PPE, about 24.0% of them indicated they used it always at the workplace. [Page 10, Line 229]
	Table 2 Pages 9, line 207-209 For the variable "use of PPE at work"; you reported as an about 355 (77.3) are not using PPE. However, in the variable "Frequency of PPE use at work" you characterize the frequency of use of PPE for 355 study participants. You are expected to characterize the 104 study participants who reported as they use PPE	Response: This has been done as suggested. It now reads as;<br><br>Out of the 104 participants that indicated they used PPE, about 24.0% of them indicated they used it always at the workplace. [Page 10, Line 229-230]
	How this can be? It is not clear are not inline. Please re consider the presentation of "use of PPE at work and Frequency of PPE use at work"Pages 10, line 230-232	We have defined how these variables were measured in methods of the revised manuscript. It reads as;<br><br>The usage of PPE was measured by asking participants whether they wore PPE at the workplace when performing a task with a 'yes' or 'no' response while the frequency of PPE usage was assessed by asking participants how often they wear the PPE when performing a task at the workplace with 'always' or 'sometimes' response. 'Always' means that the worker wears PPE anytime he or she is performing a task while 'sometimes' means that the worker only wears the PPE as and when he or she deemed it fit (i.e. not wearing it all times when performing a task at the workplace). [Page 6, Lines 152-159]
	"Workers that were working at the Port and Harbour were about 4 times (AOR: 4.32; 95%**CI: 0.14 – 16.37**) more likely to experience occupational injury compared to those at Agriculture and Forestry" The CI contains the null value (1), therefore, how this	Thank you for drawing attention to this. This has been corrected in the revised manuscript. It now reads;<br><br><br>…… port and harbour (AOR: 3.77, 95%CI: 1.70 – 8.39)…

	variable was considered as statistically significant covariate?	[Page 11, Line 243]
	Pages 11&12, line 239-241 Have you checked the assumptions of Binary logistic regression before conducting analysis? The variable" Frequency of PPE use at work" is not collected from all study participant rather only from 104 study participants; who reported as they used PPE. As result, can it can be regressed against Occupational injury?<br>Frequency of PPE use at work<br>Always 1.00 1.00<br>Sometimes 2.66 (1.43 – 4.94) 0.002 3.46 (1.42 – 8.47) 0.006<br>In the logistic regression analysis table (table 4), the outcome variable (occupational injury) with the number of injured and non-injured study participants shall be presented in a column, next to the list of variables column.	Thank you for this insightful comment. We have re-performed analysis on the logistic regression analysis again by using a backward stepwise approach. We did not include the frequency of PPE use in the model. [Page 7, Lines 181-190]
Discussion	Pages 13, line 291-293 "It is recommended that there should be reduced payment benefits to the affected worker if investigations reveal he or she was not in PPE at the time of injury." It shall be supported with credible evidence. What if the organization didn't supply adequate PPE and workers unable to purchase and use by their own?	We acknowledge your comment. The statement has been removed from the revised manuscript
	Overall comment: Please work to improve the quality of the writing throughout the manuscript	This has been done as suggested. Thank you very much
	**Reviewer 3**	
**Abstract**	Page 2 line 27, please paraphrase it.	This has been done as suggested.
	Page 2 lines 43-44: try to reanalyze and interpret the association of your outcome variable with the level of education.	This has been done as suggested. It now reads as<br><br>Tertiary education (AOR: 0.03, 95%CI: 0.01 – 0.10) and secondary education (AOR: 0.22, 95%CI: 0.07 – 0.74) were protective of occupational injuries compared to basic education. [Page 2, Lines 46-47]
	Page 2 line 45: "Working at Port 45 and Harbour (AOR: 4.32; 95%CI: 0.14 – 16.37)" Does it show a significant association?	This was an oversight and it has been corrected.<br><br>…..working at Port and Harbour (AOR: 3.77, 95%CI: 1.70 – 8.39) [Page 2, Line 43]
**Introduction**	The magnitude of the problem should be clearly stated in an inverted-triangular form (global to local perspective). The authors should clearly show the research gap that they	This has been done as suggested, please

	want to fill with the present study. They should intensively review previous research conducted in the area and direct how they will generate new evidence with their study.	
**Methods**	The study setting and area should be mentioned precisely, even with the relative and absolute location. Therefore, a map of the study area should be included	We are very grateful to the reviewer for his comments on including a map of the study area in the manuscript. However, we prefer the current state of the manuscript without the map due to copyright issues.
	Page 5 lines 118-119:<br>“All workers who reported non-occupational related 119 injuries and …… were excluded.” Do you mean all included subjects were those who have an occupational injury (Prevalence =100%)???	No please, we included all workers between 20 and 60 years. The statement portraying that we had 100% prevalence has been removed from the revised manuscript
	Page 5, line 123: why do you use a design effect of 1.2, which is nearly 1.00? What is the need to use the design effect? You have used two-stage sampling. What do you think about the proper design effect to be used for two-stage sampling? Please try to address it.	Thank you very much for your careful and meticulous observation on our manuscript. We have accepted your insightful observation on the sample size estimation relating to the design effect; the use of a design effect of 1.2 instead of 1.5. We acknowledge that this was an error and the necessary corrections have been effected in the manuscript. We apologize and will make sure that due diligence is carried out in our future submissions. [Page 5, Line 131]
	If there is more than one industrial worker in a household, how do you select the respondents? What inclusion and exclusion criteria were used during sampling?	We interviewed all industrial workers in a household, if they were more than one. This was stated in the manuscript. This is because all the workers may not be exposed to the same working conditions at their workplaces. the inclusion and exclusion criteria were workers that were 20 to 60 years and have been working in Tema for at least, one year and all workers in administrative positions (such as receptionists, accounting staff, secretaries) were excluded from the study. This has been acknowledged in the manuscript. [Page 5, Lines 124-127]
	How to control the reported bias (in case, some respondents may respond as if they were injured at the workplace, while it actually occurred in non-occupational areas)?	To reduce bias, participants who reported experiencing injuries were asked to describe how and where the injury occurred. This has been acknowledged in the revised manuscript. It now reads:<br><br>“To avoid misclassification of occupational injury, study participants who reported sustaining work-related injuries were asked to describe how and where the injury occurred. ”. [Page 6, Lines 159-162]

	Why do you use the KOBO toolbox and ODK data collection tools in combination? Use of the one will be simple for the management of the data and training of data collectors.	We have revised the statement to reflect that we used Kobo Collect for the data collection. [Page 6, Line 148]
	Some of your variables, for instance, the use of PPE, should be assessed through observation, not using a questionnaire. It raises the validity question of your finding.	We agree with the reviewer on this comment. However, we pretested the data collection instrument and had a similar result. We have acknowledged the use of self-report to assess use of PPE as a limitation in the revised manuscript. [Page 15, Lines 342-343]
**Result**	Page 10, line 231:<br><br>Please check your presentation regarding the association of level of education with occupational injuries.	This has been done as suggested. Thank you. The revised section now reads as;<br><br>Tertiary education (AOR: 0.03, 95%CI: 0.01 – 0.10) and secondary education (AOR: 0.22, 95%CI: 0.07 – 0.74) were protective of occupational injuries compared to basic education. [Page 11, Lines 246-347]
	Page 11: On tabular presentation of the logistic regression,<br>- You should include the frequency and percentage of study participants per the dichotomous outcome variable (injured/non-injured). - The P-value for the crude odds ratio is not important	Please, this was presented in Table 1 that is why we did not include it in the logistic regression table. We prefer to maintain the current state of how we presented the results in the tables. Thank you
**Discussion**	Page 12, line 249:<br>The prevalence is much higher than 50%, therefore, the phrase “more than half” is not descriptive of your finding.	This has been revised to read… The prevalence of occupational injuries (64.7%) observed in the present study was high. [Page 13, Line 262]
	The discussion and conclusions are well written	Thank you very much.

We hereby submit our revised manuscript for your consideration.

Sincerely,

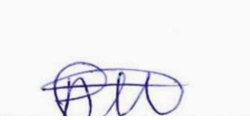

DOUGLAS ANINNG OPOKU
CORRESPONDING AUTHOR
