# Supplementary material for: Prevalence and associated factors of occupational injuries in an industrial city in Ghana
Source: PLoS One. 2024 Mar 28;19(3):e0301339. doi: 10.1371/journal.pone.0301339 (PMC10977681; doi:10.1371/journal.pone.0301339)
Supplement: Attachment — Submitted filename: RESPONSE TO REVIEWERS.pdf [file pone.0301339.s007.pdf]

ALLEN CLINIC
FAMILY HEALTHCARE SERVICES
P. O. Box X543 FNT, Kumasi, Ghana
Tel: 0322493429 | 0507171650
Loc: Ahensan Estates near KNUST Gate, Kumasi
Email: allenfhs@gmail.com

15th September, 2023

The Editor-In-Chief
PLOS One

PREVALENCE AND ASSOCIATED FACTORS OF OCCUPATIONAL INJURIES IN AN INDUSTRIAL CITY IN GHANA *(MANUSCRIPT ID.* PONE-D-23-10198R1*).*

RESPONSE TO REVIEWERS' COMMENTS

Section of paper	Reviewers' comments	Response from Authors
**Reviewer 4**		
General comments	Basically, the writing is very good, but Should be given a year limit, whether the last 10 years or the last 5, so that the prevalence data is more relevant. It just needs to be added to the background, previous research related.<br>I applaud the authors for advancing understanding and emphasizing important risk about occupational injuries<br>As mentioned in the article, understanding this subject could provide an opportunity to tackle an important public health issue in the country, especially ini occupational injuries.	We appreciate the comments of the reviewer
	Please also provide the search build document you created with the identified number of articles at each library/data base.	This is going to be extremely difficult to produce precisely because we did not set out to conduct a narrative or scoping or systematic review. This manuscript was drafted by some of the authors and the others reviewed it for critical content in addition to providing statistical support. Thus, the authors are not in a position to provide a search build document as none was deliberately created.
	Please make sure that all tables are mentioned in the main text and correctly numbered.	All the tables have been mentioned in the text as suggested
	You indicated that characteristics including sex, level of education, industry type, and occupational injuries were pooled. Though I was unable to locate the studies that were combined to create those characteristics. Please mention them.	This was a primary study and did not involve pooling of any previous data. However, we provided information on how the questionnaire was developed to collect the study data.

		The questionnaire was self-developed entirely new by the investigators through a literature review (10,21,23,33–37). Page 6, Line 164
**Reviewer 5**		
General comment	This is a cross-sectional survey of occupational injuries by industry in Ghana in 459 workers. Occupational injury rates were high, but the seriousness of these injuries were not well-described. The findings were not novel. Training programs are assumed to be important to preventing injury, but the evidence supporting this is generally weak.	We agree with the reviewer on his comments about the seriousness of the injury not described in the manuscript. Collecting and reporting data on the number of hours lost from work due to the injury would have given a better view. We have therefore acknowledged it as a study limitation in the revised manuscript.<br>The revised section now reads;<br>Again, this study did not report on how much time was lost from work due to the injury sustained at the workplace. Page 18, Line 420-421
	Education levels have been shown to be important and we assume that those with higher education better understand the importance of following safety guidelines, but education could also be capturing language barriers.	This has been captured in the discussion. The revised section now reads;<br><br>………Education could also be capturing some level of language barrier especially when most of these safety signs and guidelines are written in English which may cause a challenge in communication. Page 15, Lines 348-350
	It is also unclear what "contract engagement" means. The problem of high occupational injury rates in LMIC is a problem that needs attention.	They are casual staff and this has been replaced with 'casual staff' in the revised manuscript
	It is unclear what is meant by "safety creation awareness" in the abstract, but this might refer to safety climate or safety culture.	This has been revised to read;<br>Key preventive measures to improve safety climate at the workplace can include training in health and safety, particularly, among contract staff and those with basic or no formal education<br>Page 2, Line 49
	In terms of PPE use, is there evidence that the PPE provided to workers was functionally sound and adequate to the task? How often was it replaced? If it was worn and leaky or damaged, it would not be protective	We did not investigate that so we are unable to report on them.
	One of the most common questions asked injured workers is how much time was lost from work because this informs the seriousness of the injury. Was there a reason this question was not asked?	We agree with the reviewer on this. The question was not asked and we have acknowledged it as a study limitation in the revised manuscript. The revised section now reads;<br><br>Again, this study did not report on how much time was lost from work due to the injury sustained at the workplace.<br>Page 18, Line 420-421

	Why was sex, PPE, and monthly income not included in the multivariable model in Table 4? It was significant in the bivariate model. It seems that it would be important to control for sex and income in the model of injuries.	We have included them in the table. However, sex was included in the variables for the stepwise multivariate logistic regress. However, the Stata output did not return the variable 'sex' when adjusted for with other covariates, because it was not deemed important per our data………..likely to be the case if there was an obvious majority of males<br>Table 4, page 12
	It does not seem as if PPE would be of great value in preventing the types of injuries described in Table 2, with the exception of possibly eye injury and burns if they were to the hands. Do you think PPE is acting as a surrogate for those who are more safety-minded?	The use of PPE is crucial in injury prevention especially in situations where both administrative and engineering controls are not feasible. After adjusting for significant variables in the stepwise logistic regression, PPE use did not have any significant effect on the occurrence of injuries.
	The discussion does not make a case for specific regulations or policies that could be developed from the results of this study. How likely would it be to enforce policies around required safety and health trainings or use of PPE?	We have revised the discussion to include advocating for training and retraining of workers on health and safety at the workplace. Again, we have also advocated for the Ministry of Trade and Industry to begin taking keen interest in occupational safety. They could for instance, have an overarching unit that visits industries regularly to ascertain adherence to industry standards<br><br>Page 18, Lines 444 - 455
**Reviewer 6**		
General comment	First and foremost, I'd like to thank the authors of this research for their contribution to assessing Epidemiology of occupational injuries in an industrial city in Ghana: A cross-sectional survey. Next, I am grateful for the request to review the manuscript. In order to improve the readability of the manuscript the following points need further attention of the Authors:	Thank you and well noted. We have addressed the manuscript based on your recommendations.
Title	The title should be precise and clear. What is epidemiology for you? You said: Epidemiology of occupational injuries in an industrial city in Ghana. **Classically epidemiology is defined as:** ***The Study*** (interpreted to mean **observation, recording, testing**), **Distribution** (**P*attern*, Trends**), **Determinants** (**causes, risk factors**) of **health related states (incidence, death, treatment reaction, health care provision) and events** (not just diseases), And **Application** of this study to **detect**, **prevent** & **promotion of health** in specified populations. So, modification	We are very grateful to the reviewer on his comments about the study title. The title of the study has been revised based on the reviewer's comment.<br>The study title now reads as;<br>Prevalence and associated factors of occupational injuries in an industrial city in Ghana<br>Page 1, Line 1

	of the title should be suggested as Prevalence and associated factors of occupation-related injuries in an industrial city in Ghana.	
Introduction	The burdens and risk factors of occupation-related injuries in many occupational settings have been thoroughly established worldwide. So, what distinguishes this study from earlier research?	It is true that occupational injuries have been studied across the globe. However, in Ghana there is limited data on occupational injuries. particularly, in the Tema Metropolis, the biggest industrial setting in Ghana, no study has estimated occupational injuries. Again, this study cuts across different industries unlike the others (22, 24-26) that focused on particular industries. Estimating injury prevalence in this setting is crucial for setting priorities in promoting employees health and safety in the country.
	Additionally, there were systematic and meta-analysis findings in the African region. Justify why a primary study is needed.	This is true but the systematic review and meta-analysis did not include studies conducted in Ghana. This does NOT invalidate their findings but context-specific data are needed. Again, the particular context of Tema being an industrial hub in Ghana with limited studies relating to occupational injuries there need specific attention and hence the study.
	The authors should indicate the types of research gaps. Attention to the population gap, methodological gap, knowledge gap, empirical gap, etc.	This has been provided in the revised manuscript.<br><br>Apart from the few studies mentioned, little is known about the prevalence and factors associated with occupational injuries among workers in a major industrial city with over five hundred companies and the largest Port in Ghana. These studies only focused on estimating injury prevalence among specific working populations such as general construction workers (24), small-scale gold miners (22), healthcare workers (26), solid waste collectors (25) and Emergency Medical Technicians (27) in Ghana. To the best of the authors knowledge, no study has estimated occupational injuries among workers from different occupations in the Tema Metropolis, the biggest industrial enclave in Ghana. Lack of evidence may affect the urgency to prioritize promoting employees' health and safety in industrial cities in Ghana.<br>Page 4, Lines 100-107
	The authors tried to demonstrate the need for the research in the last introduction sections of the revised manuscript, which is appreciated.	This has been provided in the revised manuscript.

	However, the context and the need for the research should be elaborated for outsiders as well. Therefore, the authors should be expected to show the context and emphasize the novelty of the study. Scope of the research question addressed: The following factors could also play a significant role such as: The design of the working units, the structure of the working unit, and Ergonomic factors	Apart from the few studies mentioned, little is known about the prevalence and factors associated with occupational injuries among workers in a major industrial city with over five hundred companies and the largest Port in Ghana. These studies only focused on estimating injury prevalence among specific working populations such as general construction workers (24), small-scale gold miners (22), healthcare workers (26), solid waste collectors (25) and Emergency Medical Technicians (27) in Ghana. To the best of the authors knowledge, no study has estimated occupational injuries among workers from different occupations in the Tema Metropolis, the biggest industrial enclave in Ghana. Lack of evidence may affect the urgency to prioritize promoting employees' health and safety in industrial cities in Ghana.<br>Page 4, Lines 100-107
Methods	In the method section, it is good if the author(s) of this work ought to be addressing the following core issues:	We have provided them as suggested please
	the source population, study population, and study unit	The target population for this study consisted of all workers who worked and were resident in Tema Metropolis in the Greater Accra Region of Ghana<br>Page 5, Lines 135-136
	Inclusion and exclusion criteria: why peoples with age of 19 years old omitted? What is allowable age working in industry in Ghana? , justify why all workers in administrative positions (such as receptionists, accounting staff, and secretaries) were excluded from the study.	We included workers age 18 to 60 years. however, we did not get any worker below 20 years. We have clarified that in the revised manuscript. Explanations have been provided for the exclusion criteria.<br><br>The target population for this study consisted of all workers who worked and were resident in Tema Metropolis in the Greater Accra Region of Ghana. The selection of participants into the study was strictly based on satisfying the inclusion criteria which was being between 18 to 60 years (however, we did not get any worker below 20 years) old and having been working in Tema for at least twelve months. We excluded those 17 years and below because in Ghana, the minimum age of the engagement of a person that will expose him or her to hazard is 18 years (29). All workers in administrative positions (such as receptionists, accounting staff, and secretaries) were excluded

		from the study. These workers were excluded from the study because they were not directly involved in major activity at the workplace.<br><br>Page 5, Lines 135-142
	Which sampling technique is appropriate for your study? Indicate sampling techniques with a simplified schematic diagram.	Two-stage sampling technique was used to select the study participants. This was provided in the original manuscript we submitted. We have provided the diagram as an Appendix and indicated in the manuscript.<br><br>The revised section now reads;<br>Participants were recruited based on a two-stage sampling procedure with probability proportional to size (Appendix 1). In low- and middle-income countries where it is difficult to accurately record individual households, this approach was used (35,36). In the first stage of the sampling, eight (8) communities (Communities 1, 2, 3, 4, 7, 8, 9, and 10) in Tema were randomly selected from twenty-five (25) communities through balloting. In the second stage, a simple random sampling technique was used to select households in each community using a random code generator. Households that had industrial workers staying there were identified and numbered in each community. In each community, a listing was done to obtain the number of households with industrial workers. In each selected household, all eligible participants who consented were interviewed. This method was repeated in each randomly selected household in all eight communities until the estimated sample size was obtained. Page 6, Lines 148-158
	Data collection tools and techniques? Is the data collection techniques validated? If so show us the steps of tool validation.	The data collection tool was not internationally validated and this has been acknowledged in the revised manuscript. And we have also outlined the steps that were taken to improve the validity of the tool<br><br>The questionnaire was self-developed entirely new by the investigators through a literature review (10,21,23,33–37) and has not been intentionally validated. However, the study adopted robust measures such as engagement of key stakeholders (epidemiologist, biostatistician, health and safety officer, language expects etc.) for both translation

		and back-translation of the questionnaire into the local Twi language and English respectively to improve its internal validity. Page 6, Lines 162-196
	data quality assurance;	This was provided in the original manuscript under data management and statistical analysis. In the revised manuscript, we have provided further clarity and presented it as a separate subheading "Data quality assurance."<br>The revised section now reads;<br><br>Data quality assurance was conducted by the study statistician. The data was downloaded from the Kobo Collect in excel format and exported to Stata version 16.0 (StataCorp, College Station, USA) for quality management (data cleaning and coding) and analysis. The data was checked for completeness and consistency. Page 7, Lines 198-202<br><br>Again, we engaged research assistants who were final year postgraduate students who were knowledgeable in the subject area and they also underwent training on the data collection tool to improve. Page 7, Lines 183-189
	Data analysis: attention to model fitness, the model predicting ability (sensitivity and specificity), multicollinearity etc	This has been done as suggested.<br><br>A multicollinearity test was conducted to confirm the whether the explanatory variables that were included in the backward stepwise logistic regression had a correlation using the Variance Inflation Factor (VIF). The results indicated that there was no evidence of multicollinearity between the explanatory variables (mean VIF = 1.63, Maximum VIF = 2.13, Minimum VIF = 1.12) (See Appendix 1). Model fit was assessed using the Hosmer-Lemeshow test which showed that the model was good (p = 0.091). Page 8, Lines 192-224
	How was the confounding variables addressed?	We controlled for covariates that were insignificant but deemed important in the backward stepwise regression model (using a p-value of 0.1) Page 8, lines 213-215
	How validity and reliability of the finding ensured?	This was ensured by adopting an appropriate study design, and methods. The internal validity and reliability of the data collection tool were improved through pretesting and engagement of key stakeholders in developing the data collection tool as well as translation of the tool in the local Twi

		language for adequate comprehension on the part of all the study participants.<br><br>The study adopted robust measures such as pre-testing, representative sampling (two-stage sampling), engagement of key stakeholders (epidemiologist, biostatistician, health and safety officers, language experts for both translation and back-translation of the questionnaire into the local Twi language and English respectively) in developing the questionnaire. This was done to improve its reliability and internal validity.<br>Page 7, Lines 182-196
Results	What is the importance of adding a p-value to Table 1: Socio-demographic characteristics of study participants?	This has been revised and the p-value has been removed from the table
	Line 226-232: What were the dimensions of occupational health and safety measures investigated in this study? Hint: PPE utilization (as you stated), adherence to occupational safety commands, occupational health service utilization, workplace hygiene, and sanitation condition.	We investigated PPE use and frequency of use, health and safety training, availability of health and safety department as well as worker's satisfaction of health and safety measures at the workplace. This has been reported in Table 3 in the results section.
	Your study was a community based how you evaluated utilization of PPE without conducting observation whiles the employees at job?	We evaluated utilization of PPE through a self-report instead observational. This was captured under 'Data collection' in the original manuscript.<br><br>The usage of PPE was measured by asking participants whether they wore PPE at the workplace when performing a task with a 'yes' or 'no' response while the frequency of PPE usage was assessed by asking participants how often they wore the PPE when performing a task at the workplace with 'always' or 'sometimes' response. 'Always' meant that the worker wore PPE anytime he or she performed a task while 'sometimes' meant that the worker only wore the PPE as and when he or she deemed it fit (i.e. not wearing it every time a task was performed at the workplace).<br><br>Page 6, Lines 171-177<br>We are also aware of the limitation of adopting a self-report of PPE use and this was acknowledged as a study limitation under 'Strength and limitation of the study''.

		The approach of participant self-reporting of occupational injuries and the use of PPE could have affected the reported prevalence of occupational injuries and PPE use. Page 17, Lines 414-415
	How were the overall percentages of occupational health and safety practices evaluated?	We did not report on overall percentage of occupational health and safety practices. The focus of this study was to evaluate individual health and safety practices such as training, and use of PPE
	Table 3: How health and safety measures satisfaction was checked at the workplace?	This was checked by asking the study participants their overall satisfaction with health and safety measures at the workplace. This information has been provided in the data collection section of the revised manuscript.<br>The revised section now reads;<br>Data on study participants' satisfaction with existing health and safety measures at the workplace was also collected. Satisfaction with health and safety measures at the workplace was evaluated by asking the study participants their overall satisfaction with these measures with a 'yes' (meaning satisfied) or 'no' (meaning not satisfied) response. Page 6, Lines 168-173
	Line 250, attention to Table 4: associated factors of occupational injury. Saying Determinants of occupational injuries among study participants is not the appropriate word, as the design of the study was cross-sectional. I suggested factors associated with occupational injuries among study participants.	The title of Table 4 has been revised based on the reviewer's recommendation.<br>It now reads as;<br>Factors associated with occupational injuries among study participants.<br><br>Page 12, Line 289
	Why were some AORs much larger than CORs? For instances: port and harbor (AOR: 3.77, 95%CI: 244 1.70 – 8.39), no health and safety training (AOR: 2.18, 95%CI: 1.08 – 4.39) and dissatisfaction with health and safety measures. I need an explanation of it.	An explanation has been given in the revised manuscript.<br>Variables such as "monthly salary of 1000 – 1500 cedis", "ports and harbour", "pharmaceuticals" and "health and safety training" had their adjusted odds ratio in the final multivariate logistic regression model higher increased from their crude odds ratio estimates. It is possible that this may be affected by some missing data in the other variables that were considered for the final logistic regression model or a confounder. The results in this study, however, are comparable to earlier studies and offer useful information about the factors contributing to occupational injuries.<br>Page 18, Lines 421-425
	The authors should also think of effect modification**.**	We checked for effect modification using an interaction. There was evidence of interaction

		between PPE usage and gender. However, this had a minimal effect on the overall model. Page 8, Lines 217-218
	The reference categories should be included in regression table, 4.	The reference categories have been added as suggested
Discussion	The author should compare their findings with comparable populations and settings.	There were some difficulties getting comparable populations in Ghana and Africa as our study cut across many occupations instead of just one as is the case in previous studies. The closest in Africa was a study that was conducted among industrial workers in Ethiopia by Hunegnaw et al., 2021 which we compared the injury rate with in our study. Outside Africa, we compared our study with another study which was conducted in India by Sashidharan and Gopalakrishnan (2017). Page 14, Lines 323-327
	In order to improve the scientific quality of this finding, the authors should indicate what applications are there for their research findings? For example, explain how your findings enhance the general understanding of the topic to extend the reach beyond what others have found and give examples of why the world needs that increased understanding on topic under investigation.	This has been provided as suggested.
	How did you relate your study to the existing theories in the literature? You should know the existing/established theories on the subject matter & link your findings to previous theories (Whether they agree or not).	This has been done as suggested in the discussion. We have also introduced one of the theories in the study background. We also used the theory to discuss the study findings
Limitation	It could have been better if the following were also discussed: As an example, limitations could be related to Social desirability bias, Recall bias, selection bias, information bias, generalizability and transportability, variable selection, etc.	The study had some limitations. The approach of participant self-reporting of occupational injuries and the use of PPE could have affected the reported prevalence of occupational injuries and PPE use. Again, the use of self-approach could also lead to the introduction of social desirability bias. There may have been an element of recall bias since study participants had to do a retrospective (past 12 months) report of experiencing an occupational injury. However, study participants were asked to give an account of how the injury occurred to minimize the introduction of recall bias. Again, this study did not report on how much time was lost from work due to the injury sustained at the workplace. Variables such as "monthly salary of 1000 – 1500 cedis", "ports and harbour",

		"pharmaceuticals" and "health and safety training" had their adjusted odds ratio in the final multivariate logistic regression model higher increased from their crude odds ratio estimates. This may be affected by some missing data in the other variables that were considered for the final logistic regression model or a confounder. The results in this study, however, are comparable to earlier studies and offer useful information about the factors contributing to occupational injuries.<br>Despite Tema being the largest industrial hub in Ghana, data obtained from only one site limits the generalizability of the study findings. However, since the data were collected from eight communities in Tema, the study findings can be generalized to this population. Another limitation of this study was the inability to estimate the long-term injury risk (for instance, adverse health outcomes in the future from present-day inhalation of gases) of the study participants. The data collection instrument was not intentionally validated. However, pretesting, the adoption of a standard definition of occupational injury, and a simple random sampling approach were adopted to reduce biases.<br>Page 17, Lines 414-434
Conclusions	Don't restate the results. Just conclude based on your information. For instance, you said: There is a high prevalence (64.7%????) of occupational injuries among the study participants. The same is true for discussion part.	This has been done as suggested. The revised section now reads;<br>There is a high prevalence of occupational injuries among the study participants.
	You said: Future studies can adopt a qualitative approach to explore the satisfaction of health and safety policies at the workplace. Do you mean a mixed-methods approach? If so, do you think employing a qualitative approach alone is a satisfactory way to address the issue of OHS satisfaction? So, make a sound, actionable, and conclusion-based recommendation.	We have revised that statement to reflect the adoption of a mixed method approach in future studies. Both quantitative and qualitative data can be important for addressing issue of OHS.<br><br>It is imperative for effective mitigation measures to be instituted by management of these occupations for this high-risk population. These include training and retraining in health and safety, education and awareness campaigns as well as policy enforcement measures among workers, especially, those with a low level of education and temporary staff. The present study provides useful information that can guide efforts in the promotion of health and safety among workers at the workplace. The National

		Labour Commission of Ghana should also conduct routine monitoring and evaluations at various institutions (especially those in industrial cities) to ensure that employees health and safety are protected at the workplace. Future studies can adopt a mixed method approach to explore the satisfaction of health and safety policies at the workplace and how these contribute to occupational injuries. Page 18
References	Check thoroughly!	Done as suggested please.

We hereby submit our revised manuscript for your consideration.

Sincerely,

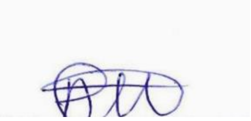

DOUGLAS ANINNG OPOKU
CORRESPONDING AUTHOR
