# Supplementary material for: Prevalence and associated factors of occupational injuries in an industrial city in Ghana
Source: PLoS One. 2024 Mar 28;19(3):e0301339. doi: 10.1371/journal.pone.0301339 (PMC10977681; doi:10.1371/journal.pone.0301339)
Supplement: Attachment — Submitted filename: AUTHORS RESPONSE.pdf [file pone.0301339.s008.pdf]

ALLEN CLINIC
FAMILY HEALTHCARE SERVICES
P. O. Box X543 FNT, Kumasi, Ghana
Tel: 0322493429 | 0507171650
Loc: Ahensan Estates near KNUST Gate, Kumasi
Email: allenfhs@gmail.com

24th February, 2024

The Editor-In-Chief
PLOS One

PREVALENCE AND ASSOCIATED FACTORS OF OCCUPATIONAL INJURIES IN AN INDUSTRIAL CITY IN GHANA *(MANUSCRIPT ID.* PONE-D-23-10198R2*).*

RESPONSE TO REVIEWERS' COMMENTS

**Section of paper**	**Reviewers' comments**	**Response from Authors**
**Reviewer 4**		
General comments	I applaud the authors for advancing understanding and emphasizing Prevalence and associated factors of occupational injuries in an industrial city in Ghana, but u must describe which industrial this research focus? manufacture industrial or?	The study adopted a multi-industry approach. We acknowledge the specificity of recommendations that come with studying specific industries but there is also a need for an overarching view of industrial injuries and their risk factors.
	Though, there are grammatical and editorial problems that need corrections.	All grammatical and editorial problems have been addressed in the revised manuscript as suggested by the reviewer
**Reviewer 6**		
General comment	The English usage is excellent and the paper is well-written. I have one remaining question and that is, why are there missing values in table 4? I am assuming there is a reason that age and gender are missing from the multivariable logistic regression results, but could not find an explanation. Could these not be estimated?	The variables 'age' and 'gender' were included in the final multivariable logistic regression analysis, but they were inherently dropped in the stepwise regression build-up by STATA.
**Reviewer 7**		
Abstract	There is no demographic data presented here. It's difficult therefore to appreciate the results since there is no data on demography.	We have presented some demographic variables in the results of the abstract as suggested by the reviewer.<br><br>*The mean age of the workers was 33.9 (±6.8) years with a range of 21 – 53 while over 18.1% of them were working at the Port and Harbour*
Abstract	The conclusion is vaguely written and does not seem to be supported by the results.	The conclusion has been revised to reflect the results as suggested by the reviewer. The revised section now reads as;

		The prevalence of occupational injuries in this study was high. Promoting machine tools' safety, health and safety training, and satisfaction with health and safety measures through rewarding workers who do not sustain injuries could be key to employees' health and safety.
Introduction	Line 79: is it ‘former’?	This is an error, it is ‘former.’ This has been corrected in the revised manuscript<br>*…who rely on the former to take action*
Discussion	Line 378-382- This seems to be mere speculation. Please back it up with references.	The statement has been revised with a reference provided. It now reads as;<br>*Casual workers are mostly inexperienced, less knowledgeable about occupational hazards and usually do not benefit from health and safety training at the workplace, predisposing them to occupational injuries (55,56)*
Discussion	Line 400-401- This is mere speculation. Please back it up with references. It’s obvious that workers with low pay may be prone to occupational injuries as supported my many studies. Please get some concrete reasons backed by literature as to why you had these findings	We appreciate the comment made by the reviewer. The statement in reference has been taken off the revised manuscript
Conclusion	The conclusion should be short and concise and focus on the major findings from the work. Line 442 to 455 should be deleted. These are recommendations that are not part of conclusion.	The conclusion has been revised and Lines 442 to 445 have been deleted as suggested by the reviewer.<br><br>*There is a high prevalence of occupational injuries among the study participants and these were mainly caused by working tools and hot surfaces, substances or chemicals. Factors such as employees’ level of education, type of engagement, monthly income, type of industrial work, health and safety training and satisfaction with health and safety measures at the workplace were independently associated with occupational injuries. The Ministry of Trade and Industry, Department of Factories Inspectorate, and National Labour Commission of Ghana should collaborate and conduct routine monitoring and evaluations at various institutions (especially those in industrial cities) to ensure that employees' health and safety are protected at the workplace. We also recommend that the Association of Ghana Industries (AGI) should create more awareness of employees’ health and safety by admonishing their members to be conscious of the need to adhere to safety practices at the workplace. Future studies*

		can adopt a mixed-method approach to explore the satisfaction of health and safety policies at the workplace and how these contribute to occupational injuries.

We hereby submit our revised manuscript for your consideration.

Sincerely,

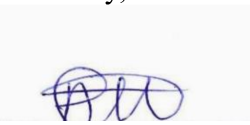

DOUGLAS ANINNG OPOKU
CORRESPONDING AUTHOR
