# Supplementary material for: Prevalence and associated factors of occupational injuries in an industrial city in Ghana
Source: PLoS One. 2024 Mar 28;19(3):e0301339. doi: 10.1371/journal.pone.0301339 (PMC10977681; doi:10.1371/journal.pone.0301339)
Supplement: Attachment — Submitted filename: Reviewer plos one.docx [file pone.0301339.s004.docx]

**Reviewer’s comments**

Introduction

Page 3, line 61

“The global burden of occupational injuries is estimated to be 1.9 million deaths in 2019” is this data/figure includes occupational diseases OR occupational injuries only?

Pages 4, line 88-92

The statement is about the associated factors; as result it is better to merge with the previous paragraph.

Pages 4, line 93

“Apart from the few studies mentioned above,…”

What your study added to this few studies?

Methods

Pages 5, line 123

Why you use 1.2 as design effect? Why not 1.5 or 2?

Could you support with credible source/evidence

Pages 6, line 150-152

“’Occupational injury’ was operationalized as all injuries sustained while performing a task at the workplace that resulted in a loss of at least 12 hours of productive time”

How did you confirm the impact of injury (a loss of at least 12 hours of productive time)?

Pages 6, line 160-161

Are you used Chi square or Fisher’s exact tests analysis? Better to state what you already used

Results

Pages 7, line 185-186

“The proportion of study participants that experienced an occupational injury in the last 12 months

was 64.7%.”

State the proportion of occupational injuries with 95% CI; the lower and upper confidence intervals

Pages 8, line 197-199

“Over 54.9% of them were supplied with PPE and 77.3% of the study participants indicated that they used PPE at the workplace”

How the proportion of PPE use is much higher than the proportion of PPE supply (77.5% vs 54.9%)

Table 2

Pages 9, line 207-209

For the variable “use of PPE at work”; you reported as an about 355 (77.3) are not using PPE. However in the variable “Frequency of PPE use at work” you characterize the frequency of use of PPE for 355 study participants. You are expected to characterize the 104 study participants who reported as they use PPE

How this can be? It is not clear are not inline. Please re consider the presentation of “use of PPE at work and Frequency of PPE use at work”

Pages 10, line 230-232

“Workers that were working at the Port and Harbour were about 4 times (AOR: 4.32; 95%**CI: 0.14 – 16.37**) more likely to experience occupational injury compared to those at Agriculture and Forestry”

The CI contains the null value (1), therefore, how this variable was considered as statistically significant covariate?

Pages 11&12, line 239-241

Have you checked the assumptions of Binary logistic regression before conducting analysis?

The variable” Frequency of PPE use at work” is not collected from all study participant rather only from 104 study participants; who reported as they used PPE. As result, can it can be regressed against Occupational injury?

In the logistic regression analysis table (table 4), the outcome variable (occupational injury) with the number of injured and non-injured study participants shall be presented in a column, next to the list of variables column.

Discussion

Pages 13, line 291-293

“It is recommended that there should be reduced payment benefits to the affected worker if investigations reveal he or she was not in PPE at the time of injury.”

It shall be supported with credible evidence. What if the organization didn’t supply adequate PPE and workers unable to purchase and use by their own?

Overall comment

Please work to improve the quality of the writing throughout the manuscript
