# Supplementary material for: Prevalence and associated factors of occupational injuries in an industrial city in Ghana
Source: PLoS One. 2024 Mar 28;19(3):e0301339. doi: 10.1371/journal.pone.0301339 (PMC10977681; doi:10.1371/journal.pone.0301339)
Supplement: Attachment — Submitted filename: A review results, INJURY.docx [file pone.0301339.s006.docx]

First and foremost, I'd like to thank the authors of this research for their contribution to assessing Epidemiology of occupational injuries in an industrial city in Ghana: A cross-sectional survey. Next, I am grateful for the request to review the manuscript. In order to improve the readability of the manuscript the following points need further attention of the Authors:

1. **Title**

- The title should be precise and clear. What is epidemiology for you? You said: Epidemiology of occupational injuries in an industrial city in Ghana. **Classically epidemiology is defined as: *The Study*** (interpreted to mean **observation, recording, testing)**,  **Distribution** (**P*attern*, Trends)**,  **Determinants** (**causes, risk factors**) of **health related states (incidence, death, treatment reaction, health care provision) and events** (not just diseases), And **Application** of this study to **detect**, **prevent & promotion of health** in specified populations.
- So, modification of the title should be suggested as Prevalence and associated factors of occupation-related injuries in an industrial city in Ghana.

2. **Introduction**

- The burdens and risk factors of occupation-related injuries in many occupational settings have been thoroughly established worldwide. So, what distinguishes this study from earlier research?
- Additionally, there were systematic and meta-analysis findings in the African region. Justify why a primary study is needed.
- The authors should indicate the types of research gaps. Attention to the population gap, methodological gap, knowledge gap, empirical gap, etc.
- The authors tried to demonstrate the need for the research in the last introduction sections of the revised manuscript, which is appreciated. However, the context and the need for the research should be elaborated for outsiders as well.
- Therefore, the authors should be expected to show the context and emphasize the novelty of the study.
- Scope of the research question addressed:
  •  The following factors could also play a significant role such as: The design of the working units, the structure of the working unit, and  Ergonomic factors.

3. **Method section**

- In the method section, it is good if the author(s) of this work ought to be addressing the following core issues:
- the source population, study population, and study unit
- Inclusion and exclusion criteria: why peoples with age of 19 years old omitted? What is allowable age working in industry in Ghana? , justify why all workers in administrative positions (such as receptionists, accounting staff, and secretaries) were excluded from the study.
- Which sampling technique is appropriate for your study? Indicate sampling techniques with a simplified schematic diagram.
- Data collection tools and techniques
  - Is the data collection techniques validated? If so show us the steps of tool validation.
- data quality assurance;
- Data analysis: attention to model fitness, the model predicting ability(sensitivity and specificity), multicollinearity etc
- How was the confounding variables addressed?
- How validity and reliability of the finding ensured?

4. **Results**

- What is the importance of adding a p-value to Table 1: Socio-demographic characteristics of study participants?
- Line 226-232: What were the dimensions of occupational health and safety measures investigated in this study? Hint: PPE utilization (as you stated), adherence to occupational safety commands, occupational health service utilization, workplace hygiene, and sanitation condition.
- Your study was a community based how you evaluated utilization of PPE without conducting observation whiles the employees at job?
- How were the overall percentages of occupational health and safety practices evaluated?
- Table 3: How health and safety measures satisfaction was checked at the workplace?
- Line 250, attention to Table 4: associated factors of occupational injury. Saying Determinants of occupational injuries among study participants is not the appropriate word, as the design of the study was cross-sectional. I suggested factors associated with occupational injuries among study participants.
  - Why were some AORs much larger than CORs? For instances: port and harbor (AOR: 3.77, 95%CI: 244 1.70 – 8.39), no health and safety training (AOR: 2.18, 95%CI: 1.08 – 4.39) and dissatisfaction with health and safety measures. I need an explanation of it.
  - The authors should also think of effect modification**.**
- The reference categories should be included in regression table, 4.

5. **Discussion**

- The author should compare their findings with comparable populations and settings.
- In order to improve the scientific quality of this finding, the authors should indicate what applications are there for their research findings? For example, explain how your findings enhance the general understanding of the topic to extend the reach beyond what others have found and give examples of why the world needs that increased understanding on topic under investigation.
- How did you relate your study to the existing theories in the literature?
- You should know the existing/established theories on the subject matter & link your findings to previous theories (Whether they agree or not).

Limitation:

- It could have been better if the following were also discussed: As an example, limitations could be related to Social desirability bias, Recall bias, selection bias, information bias, generalizability and transportability, variable selection, etc.

6. **Conclusions**

- Don’t restate the results. Just conclude based on your information. For instance, you said: There is a high prevalence (64.7%????) of occupational injuries among the study participants. The same is true for discussion part.
- You said: Future studies can adopt a qualitative approach to explore the satisfaction of health and safety policies at the workplace. Do you mean a mixed-methods approach? If so, do you think employing a qualitative approach alone is a satisfactory way to address the issue of OHS satisfaction?
- **So, make a sound, actionable, and conclusion-based recommendation.**

7. **References.**

- **Check thoroughly!**
